# An Attention-Based CoT-ResNet With Channel Shuffle Mechanism for Classification of Alzheimer’s Disease Levels

**DOI:** 10.3389/fnagi.2022.930584

**Published:** 2022-07-11

**Authors:** Chao Li, Quan Wang, Xuebin Liu, Bingliang Hu

**Affiliations:** ^1^Key Laboratory of Spectral Imaging Technology, Xi’an Institute of Optics and Precision Mechanics, Chinese Academy of Sciences, Xi’an, China; ^2^University of Chinese Academy of Sciences, Beijing, China; ^3^Key Laboratory of Biomedical Spectroscopy of Xi’an, Xi’an Institute of Optics and Precision Mechanics, Chinese Academy of Sciences, Xi’an, China

**Keywords:** Alzheimer’s disease, MRI, CoT module, Channel Shuffle, ResNet, medical image classification

## Abstract

Detection of early morphological changes in the brain and early diagnosis are important for Alzheimer’s disease (AD), and high-resolution magnetic resonance imaging (MRI) can be used to help diagnose and predict the disease. In this paper, we proposed two improved ResNet algorithms that introduced the Contextual Transformer (CoT) module, group convolution, and Channel Shuffle mechanism into the traditional ResNet residual blocks. The CoT module is used to replace the 3 × 3 convolution in the residual block to enhance the feature extraction capability of the residual block, while the Channel Shuffle mechanism is used to reorganize the feature maps of different groups in the input layer to improve the communication between the feature maps from different groups. Images of 503 subjects, including 116 healthy controls (HC), 187 subjects with mild cognitive impairment (MCI), and 200 subjects with AD, were selected and collated from the ADNI database, and then, the data were pre-processed and sliced. After that, 10,060 slices were obtained and the three groups of AD, MCI and HC were classified using the improved algorithms. The experiments showed that the refined ResNet-18-based algorithm improved the top-1 accuracy by 2.06%, 0.33%, 1.82%, and 1.52% over the traditional ResNet-18 algorithm for four medical image classification tasks, namely AD: MCI, AD: HC, MCI: HC, and AD: MCI: HC, respectively. The enhanced ResNet-50-based algorithm improved the top-1 accuracy by 1.02%, 2.92%, 3.30%, and 1.31%, respectively, over the traditional ResNet-50 algorithm in four medical image classification tasks, demonstrating the effectiveness of the CoT module replacement and the inclusion of the channel shuffling mechanism, as well as the competitiveness of the improved algorithms.

## Introduction

Alzheimer’s disease (AD) is the most common degenerative neurological disease among the elderly, accounting for approximately 80% of all dementia subjects, and is the sixth leading cause of death in the United States (Dunn et al., 2021). According to the level of cognitive impairment, there are also conditions that have not been diagnosed as AD, namely healthy controls (HC) and moderate cognitive impairment (MCI). Among them, MCI is a common cognitive decline disorder, which is a transitional state between HC and AD (Mimura et al., 2021). Around the world, more than 55 million people are estimated to have dementia, and the number of people affected will increase to 139 million by 2050 (Kuehn, 2021). Although many clinical trials of drug candidates are now underway, there are few effective therapeutic agents for AD (Yamamoto et al., 2022). Early diagnosis and intervention of AD have the potential to delay or slow down the disease from progressing, so early diagnosis studies of AD are of great importance (Laske et al., 2015).

In recent years, magnetic resonance imaging (MRI) has been valuable in evaluating AD patients. We have identified it as the imaging method for many clinical conditions. An important feature of AD is progressive brain atrophy, which can be detected with the help of high-resolution quantitative MRI scanning techniques that can examine changes in brain anatomy in vivo and identify areas that are affected in the early stages of AD (Chan et al., 2001). However, manual analysis and processing of MRI images have the disadvantages of being subjective and time-consuming. Algorithm-based computer-aided diagnosis can better classify medical images, which can help doctors improve diagnosis efficiency. Deep learning technology has excellent learning ability for images, and it has great advantages in the processing and analysis of complex brain neuroimaging. MRI voxel points were used as features (Salvatore et al., 2015), followed by principal component analysis (PCA) to reduce the complexity of the data, which was then fed into a support vector machine (SVM) (Hearst et al., 1998) to train a classification model that achieved an accuracy of 76% for AD vs. HC. An SVM is a weak classifier, and multiple weak classifiers can be integrated into one strong classifier by Adaboost (Hastie et al., 2009) algorithm to further improve the accuracy of classification. Bilateral hippocampal volumes and low-frequency amplitude values with significant differences in all groups of brain regions were selected as classification features (Falahati et al., 2014), and the MRI data of AD, MCI, and HC were classified in pairs using the Adboost integration method, and the accuracy of AD vs. HC classification could reach 78.57%. However, the above-mentioned studies required many complex pre-processing tasks, which were time-consuming and did not provide high accuracy in classification. Because traditional machine learning algorithms are sensitive to data. However, deep learning techniques alleviate this problem by not requiring many pre-processing steps on the data, and just feeding the simply processed data to the algorithmic model, which can be trained to learn the features of the data and can classify and identify AD (Ding et al., 2019; Sharma et al., 2020; Suh et al., 2020). A deep learning-based cascaded autoencoder for feature representation (Suk and Shen, 2013), which combined latent information such as nonlinear relationships with original low-level features, helped to construct a robust model for AD: MCI classification with high diagnostic accuracy, and experiments conducted on the ADNI dataset showed that the accuracy of the method for AD and MCI diagnosis was 95.9% and 85.0%, respectively. To effectively mitigate the over-fitting of the network, the dropout method provided a simple technique to avoid the over-fitting in feed-forward neural networks. A robust deep learning system to identify different progression stages of AD patients based on MRI and positron emission tomography (PET) scans (Li et al., 2014), using dropout techniques, improved classical deep learning algorithms by preventing co-adaptation of weights, with an average improvement of 6.2% in classification accuracy. The fusion of traditional machine learning algorithms with deep learning also has some advantages. Deep Belief Network (DBN) was used for MRI and PET images (Ortiz et al., 2016), and the DBN automatically extracted the high-dimensional features for the training of the support vector machine. The high-dimensional features extracted from the last convolution layer of the CNN were flattened into a one-dimensional vector and fed into a fully connected network with a SoftMax classifier to obtain an AD vs. HC classifier for AD-aided diagnosis. In recent years, the algorithmic framework of transfer learning has also been applied to image classification problems. The early stages of AD were diagnosed by using hierarchical transfer learning and tissue segmentation of brain images (Mehmood et al., 2021). The proposed model outperformed the latest models in testing accuracy in layer-by-layer transfer learning using VGG (Simonyan and Zisserman, 2014) architecture with pre-trained weights for classification.

Machine learning techniques, especially deep learning techniques, are driving innovation in AD recognition and classification tasks. However, the problems of a large number of model parameters, the difficulty in training the models, and the unsatisfactory accuracy of AD recognition are still significant. Therefore, finding a deep learning classification method with a few parameters and efficient to train is substantial for the recognition and classification of AD.

In this paper, two improved image classification network models based on ResNet-18 and ResNet-50 (He et al., 2016) are proposed. The two algorithms introduce a self-attention mechanism (Vaswani et al., 2017) and partial convolution layer to extract global and local features of the input information, respectively, and add group convolution and Channel Shuffle mechanism (Zhang et al., 2018) to the ResNet-50 module, which can effectively improve the model’s global and local information attention, enhance the feature extraction ability of residual blocks, and improve the classification accuracy for different levels of AD and HC.

## Related Work

### Residual Neural Network

There have been a series of breakthroughs in computer vision in the past few years. In particular, introducing deep convolution neural networks has achieved many advanced results in image classification and recognition problems. Therefore, many researchers prefer to use deeper neural networks to solve more complex tasks and improve the accuracy of classification and recognition by adding more layers to the network. However, as the number of layers of neural networks continues to deepen, training becomes difficult, and accuracy decreases. Deep Residual Network (ResNet) is a specific type of neural network proposed by He et al. (2016) further to deepen the number of layers of the network, and the model won first place in the ILSVRC 2015 classification competition with a top-5 error rate of 3.57%. ResNet emerged mainly to solve the complex problem of stacking residual blocks in deep neural networks, thus improving the network’s accuracy and performance. By introducing residual blocks, the issue of training very deep networks is eased. ResNet skips some intermediate layers, called skip connections, which are the essence of residual blocks, unlike traditional neural networks.

The residual block in ResNet comprises a weight layer and a ReLU function (Agarap, 2018). When the input is *x*, the learned feature is denoted as *H*(*x*), and the residual part is *F*(*x*) = *H*(*x*)−*x*. The stacked layers further learn new features based on the input features. The network has better performance, and even if the learning in the network is 0, even when the residual knowledge is 0, the identity mapping will not cause the network performance to degrade. From a mathematical point of view, it can express the learning of the residual block as:


$$y_{l}=h\,\left(x_{l}\right)+F\,\left(x_{l},W_{l}\right)$$

$$x_{l+1}=f\,\left(y_{l}\right)$$

Among them, the input and output of the *l*-th residual block unit are *x*_*l*_ and *x*_*l* + 1_, respectively, the learned residual is denoted as *F*, *h*(*x*_*l*_) = *x*_*l*_ represents the identity mapping, and *f* represents the ReLU activation function, which can be obtained from the shallow layer of the network. The features learned from *l* to deep *L* can be expressed as:


$$x_{L}=x_{l}+\sum_{i=l}^{L-1}F\,\left(x_{i},W_{i}\right)$$

The gradient back-propagation (LeCun et al., 1988) process for ResNets can be obtained using chain derivation rules:


$$\frac{\partial l\,o\,s\,s}{\partial x_{l}}=\frac{\partial l\,o\,s\,s}{\partial x_{L}}\cdot \frac{\partial x_{L}}{\partial x_{l}}=\frac{\partial l\,o\,s\,s}{\partial x_{L}}\cdot \left(1+\frac{\partial }{\partial x_{L}}\,\sum_{i=l}^{L-1}F\,\left(x_{i},W_{i}\right)\right)$$

The gradient generated by the loss function reaching the deep layer of the network is $\frac{\partial l\,o\,s\,s}{\partial x_{L}}$, and the gradient generated by the constant mapping is 1. The existence of “1” can ensure that the gradient will not disappear during the back-propagation, so the residual block can be used to deepen the level of the network and learn more features. It should be noted that the number of channels of some 3 × 3 convolutional layers in the residual block has not changed.

### Contextual Transformer

Inspired by Transformer’s self-attention mechanism in natural language processing, many researchers have explored the application of the self-attention mechanism in computer vision task scenarios. By the combination of attention mechanism, it can not only verify the judgment basis of the deep learning model, but also make the deep learning model pay more attention to the important features in order to improve the performance of the deep learning model. In addition, the attention mechanism can be effectively used in medical image analysis, and its application to medical image processing has a good prospect.

**FIGURE 1:**
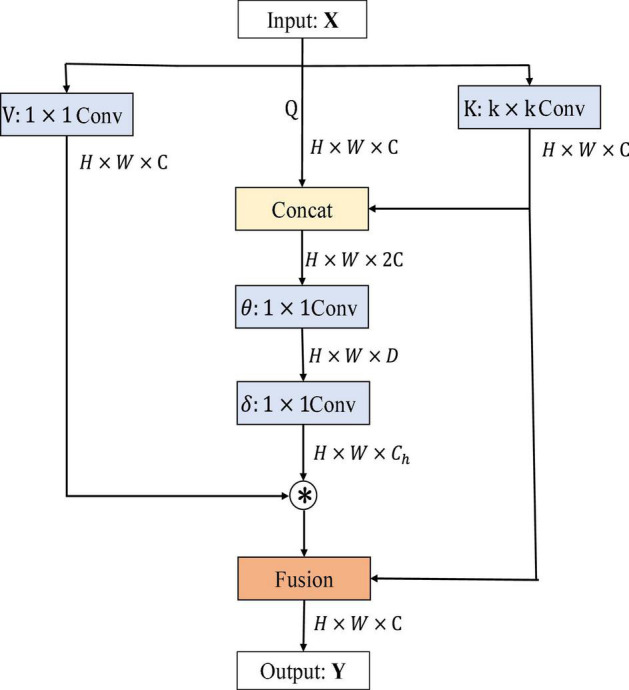
Contextual Transformer (CoT) block architecture.

First, channel-based attention or non-local relations across images add self-attention to the convolution neural network. Second, the extraction of visual features by the convolution neural network is enhanced by replacing some convolution layers with self-attention layers, thus improving image classification and detection. Third, it combines the attention mechanism with convolution features and achieves excellent results. Under the same computational cost and model size constraints, the architecture of the attention mechanism can achieve competitive image classification accuracy. Traditional self-attention mechanisms interact based on input obtained feature information across different spatial locations, but all paired query–key relationships are independently learned on isolated query–key pairs without exploring the context between their transmission. It can fuse rich contextual information and play a huge part in visual representation of 2D images. Therefore, a new attention mechanism–contextual transformation module is considered to be added to ResNet. It showed the architecture of the contextual transformer (CoT) module (Li et al., 2022) in Figure 1. The CoT module combines the advantages of Transformer and CNN, where Transformer can get the global information of input features and CNN can capture the local knowledge of input features, combining the advantages of each to improve the feature representation of input information by the network model. It showed the architecture of the CoT module in Figure 1.

*H*, *W*, and *C* denote the height, width, and number of channels of the input data *X*, *D* is the change value of the channel, *C_h_* is the header number, *Q*, *K*, and *V* represent queriers, keys, and values respectively, θ and δ denote 1 × 1 convolution operations, ⊗ denotes matrix multiplication, and *W_v_* denotes embedding matrix. Firstly, for the input feature *X*, three variables, namely, *Q* = *X*, *K* = *X*, *V* = *XW*_*v*_, are defined. A convolution of *k* × *k* is performed on *K* to get *K* with local contextual information representation, denoted as *K*^1^, which can be regarded as static modeling of local information. Then, *K*^1^ and *Q* were concatenated, and then, two successive convolution operations were performed on the result of the concatenation.


$$A=\left[K^{1},Q\right]\,W_{\theta }\,W_{\delta }$$

where *A* matrix is got from the interaction of query information and local context information, rather than just modeling the relationship between query and key. It is the self-attention mechanism that is enhanced by the guidance of local context modeling *K*^1^. Finally, this attention map was multiplied by V to get the dynamic context modeling *K*^2^. Finally, the result of CoT is the fusion of *K*^1^ for local static context modeling and *K*^2^ for global dynamic context modeling.

Overall, the input image data dimensions are first convolved by *k* × *k* convolution kernel to obtain the local information of the image, and then, the local information is spliced and fused with the original input information, so that the number of channels of output features becomes twice that of the original. Secondly, the attention matrix is obtained by two convolution kernels, and then, the matrix product operation is performed with the output result of convolution kernel operation to obtain the local and global information of the image. Finally, the local features extracted by CNN and the global features extracted by self-attention are added and fused to obtain the output feature *Y*. It is worth noting that the input dimension of the CoT module is consistent with the output dimension, and the number of channels has not changed. Therefore, it can be embedded into the residual block of ResNet.

### Channel Shuffle Structure

**FIGURE 2:**
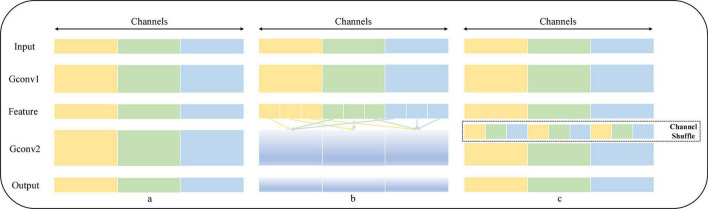
Schematic diagram of the operation of Channel Shuffle.

Channel Shuffle is an operation that helps information flow across channels in a convolution neural network. If a group convolution is allowed to take input data from different groups, the input and output channels will be correlated, which allows information to be communicated interactively between different groups. Specifically, for the feature map generated by the upper layer, the channels in each group can be divided into several subgroups, and then, different subgroups can be transferred to each group in the next layer, so the information exchange between subgroups of group convolution is strengthened. The operation process of Channel Shuffle is shown in Figure 2. Figure 2A shows an ordinary group convolution operation, which isolates all the operations, resulting in the output into associating a very small part of the input information, resulting in the result that the information between groups cannot be exchanged, thus reducing the expressiveness of the input; Figure 2B shows a random disruption reassignment for the dimension of the output, which is actually uniform disruption operation, dividing the output of each group into multiple subgroups and then inputting multiple subgroups into different groups in order to realize that the information between groups can be retained; and in Figure 2C, the feature map of the output in Figure 2B is reorganized, and the input of the next layer is from different, ensuring that the information can be interactively circulated between different groups.

## Algorithm Design

### The First Algorithm

The first algorithm takes the ResNet-18 structure as the backbone network. The main idea is to replace a convolution block in the module of ResNet-18 with a CoT module. In convolution operation, because of the small receptive field of the convolution kernel, the output feature information extracted through the convolution kernel is limited. At the same time, the self-attention mechanism can get global information through the larger receptive field. Therefore, introducing the CoT module for ResNet means introducing the contextual information of self-attention mechanism for ResNet-18 and combining the local contextual information extracted by convolution to effectively fuse the two and improve the visual representation of ResNet.

**FIGURE 3:**
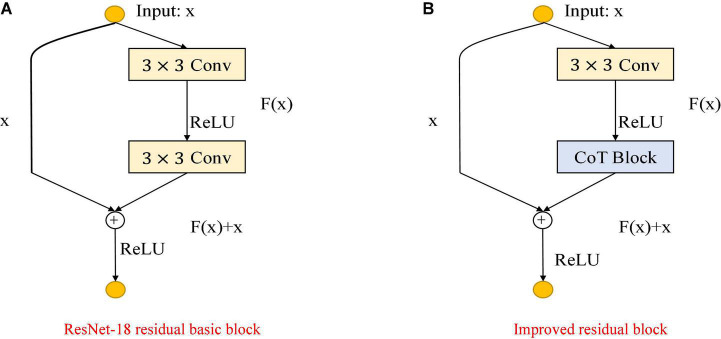
Comparison of the improved ResNet-18 residual block. **(A)** ResNet-18 residual block. **(B)** Improved residual block.

**FIGURE 4:**
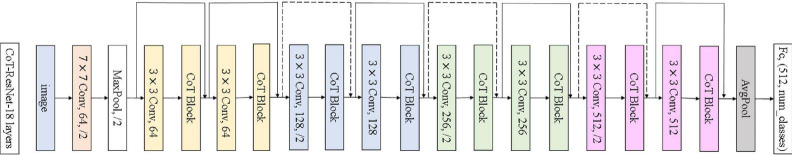
CoT-ResNet-18 architecture diagram.

As shown in Figure 3, the improvement in the module is mainly to use the CoT module to replace the convolution block in the original residual block and to improve the feature expression ability of the residual block to the input data. The left figure is the original residual block, containing two convolution layers and a skip connection. The output of the first convolutional layer is activated using the ReLU function, and then the input is added directly before the final ReLU activation function by skipping two convolutional operations. Output features are output as the extracted features. The right figure shows the improved residual block, replacing the convolution block in the original residual block with the CoT module, introducing an attention mechanism to the ResNet, and further enhancing the feature representation and performance of the algorithm. The structure of the improved CoT-ResNet-18 is shown in Figure 4, and the comparison between ResNet-18 and CoT-ResNet-18 is shown in Table 1.

**TABLE 1:** Comparison of ResNet-18 and CoT-ResNet-18.

Layer name	Output size	ResNet-18	CoT-ResNet-18
Conv1	112×112	7×7 64 stride 2	7×7 64 stride 2
Conv2.x	56×56	3×3 max pool stride 2	3×3 max pool stride 2
		[3×3 conv2d, 643×3 conv2d, 64]×2	[3×3 conv2d, 64 CoT,64]×2
Conv3.x	28×28	[3×3 conv2d, 1283×3 conv2d, 128]×2	[3×3 conv2d, 128 CoT, 128]×2
Conv4.x	14×14	[3×3 conv2d, 2563×3 conv2d, 256]×2	[3×3 conv2d, 256 CoT, 256]×2
Conv5.x	7×7	[3×3 conv2d, 5123×3 conv2d, 512]×2	[3×3 conv2d, 512 CoT, 512]×2
	1×1	*averagepool*, 1000−d *fc*, *softmax*	*averagepool*, 1000−d *fc*, *softmax*

As shown in Figure 4, the 3 × 3 convolution blocks in the original architecture of ResNet-18 are replaced with the CoT module, which can further enhance the feature extraction capability of the network for the input data. Each residual block can learn the global and local information of the input features using the self-attention mechanism and convolution operation in the CoT module. Then, the global and regional information can be fused, enhancing the network model’s ability to represent the input features and thus improve the performance based on the original ResNet-18. The network’s input is a 224 × 224 2D image, which goes through multiple residual blocks and a final fully connected layer to achieve disease classification and prediction.

### The Second Algorithm

The second algorithm takes the ResNet-50 structure as the backbone and improves based on the residual block. The CoT module is used to replace the 3 × 3 convolution blocks in the original residual blocks, the 1 × 1 convolution blocks are changed to group convolution, and then, the grouped output feature maps are channel shuffled. The primary purpose is to enhance the feature extraction ability of the data using the CoT module and then reduce the number of parameters of the model using group convolution to enhance the information communication between different groups to achieve the purpose of improving the classification accuracy and slightly reducing the number of model parameters. A learnable grouped convolution is used in ResNet-50 to replace the normal convolutional layers, allowing flexible grouping structures and yielding better representation capabilities. The grouped convolution approach allows for a better trade-off between accuracy and speed than normal convolution.

**FIGURE 5:**
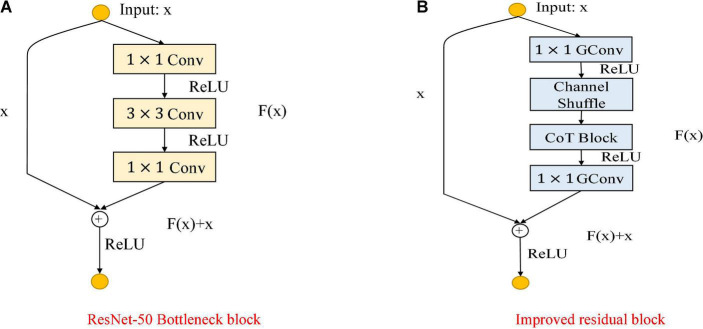
Comparison of the improved ResNet-50 residual block. **(A)** ResNet-50 residual block. **(B)** Improved residual block.

**FIGURE 6:**
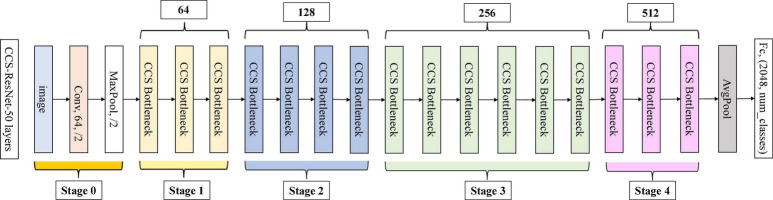
CCS-ResNet-50 architecture diagram.

As shown in Figure 5, the Figure 5A shows the bottleneck block of ResNet-50, which contains two 1 × 1 convolution layers with the main purpose of changing the number of channels and 3 × 3 convolution layers for extracting features from the input information; the Figure 5B shows the improved residual block, whose basic structure is similar to that of the bottleneck block. Improvements include replacing the 1×1 convolution layers in the bottleneck block with 1×1 group convolution layers, and adding the Channel Shuffle mechanism to randomly reorganize the output features after the group convolution layers to enhance the information exchange of the output features from different groups. The improved residual block shown in Figure 5B is named CoT with Channel Shuffle Bottleneck (CCS Bottleneck). The enhanced CCS-ResNet-50 model architecture is shown in Figure 6, and the comparison between ResNet-50 and CCS-ResNet-50 is shown in Table 2.

**TABLE 2:** Comparison of ResNet-50 and CCS-ResNet-50.

Layer name	Output size	ResNet-50	CCS-ResNet-50
Conv1	112×112	7×7 64 stride 2	7×7 64 stride 2
Conv2.x	56×56	3×3 max pool stride 2	3×3 max pool stride 2
		[1×1 conv2d, 643×3 conv2d, 641×1 conv2d, 256]×3	[1×1 Gconv2d, 64 channel Shuffle CoT, 641×1 Gconv2d, 256]×3
Conv3.x	28×28	[1×1 conv2d, 1283×3 conv2d, 1281×1 conv2d, 512]×4	[1×1 Gconv2d, 128 channel Shuffle CoT, 1281×1 Gconv2d, 512]×4
Conv4.x	14×14	[1×1 conv2d, 2563×3 conv2d, 2561×1 conv2d, 1024]×6	[1×1 Gconv2d, 256 channel Shuffle CoT, 2561×1 Gconv2d, 1024]×6
Conv5.x	7×7	[1×1 conv2d, 5123×3 conv2d, 5121×1 conv2d, 2048]×3	[1×1 Gconv2d, 512 channel Shuffle CoT, 5121×1 Gconv2d, 2048]×3
	1×1	*averagepool*, 1000−d *fc*, *softmax*	*averagepool*, 1000−d *fc*,*softmax ^f^*

As shown in Figure 6, the CCS-ResNet-50 architecture is divided into five stages, of which Stage 0 has a simple structure and can be regarded as the pre-processing of inputs, while the last four stages are composed of CCS Bottleneck and have a similar structure. The remaining three stages include 4, 6, and 3 CCS Bottleneck, respectively, and finally, the output is implemented via average pooling and full connection.

### Loss Function

The cross-entropy (Zhang and Sabuncu, 2018) loss function is used for the training of the improved ResNet. For the multi-classification task, the cross-entropy loss function is that,


$$\text{loss}=-\sum_{c=1}^{M}y_{o,c}\,\log\left(p_{o,c}\right)$$

where *M* is the total number of ADs with different levels, *p*_*o*,*c*_ is the probability value that the model determines that the observation item *o* belongs to class c, and it is a binary indicator (0 or 1). If the observation item *o* can be correctly classified as c, the value of *y*_*o*,*c*_ is 1; otherwise, it is 0. When performing batch training to calculate the loss, the cross-entropy loss function is used to evaluate the difference between the probability distribution of the model’s current training and the true data distribution. The smaller the value calculated by the cross-entropy loss function, the closer the two distributions are.

### Experimental Parameter Setting

This paper has implemented the two proposed algorithms using the commonly used machine learning library PyTorch 1.9.0+cu102 and Python 3.6 for building the network module. Hardware platforms used for the experiments are as follows: CPU is Intel(R) Xeon(R) Silver 4214, GPU is NVIDIA GeForce RTX 3090, and a video memory size is 48G. The processed MRI dataset contains 10,060 sliced images containing 4,000 AD slices, 3,740 MCI slices, and 2,320 HC slices. The 10,060 slices were randomly disorganized. The training, validation, and test datasets were split into 8:1:1, and the accuracy of the AD:HC, AD:MCI, MCI:HC, and AD:HC:MCI tasks was evaluated to examine the performance of the model classification. In the model training process, the experimental loss function is the cross-entropy loss function, and the model optimization uses the Adam optimizer (Kingma and Ba, 2014), with the initial learning rate set to 3e-5 and the batch size set to 32. The number of training epochs is set to 100, and if the model is failed to improve in every 10 training epochs, the learning rate is reduced by 10 times, and if the training loss does not decrease after 30 epochs, the training is stopped to prevent over-fitting. The models are trained and learned for 10 epochs, the performance of the models is tested on the validation set, the models with the highest accuracy on the validation set are saved, and when all training is complete, the final classification accuracy is tested on the test set. For the evaluation criteria of the experiments, we used the top-1 accuracy, precision, and recall, which are commonly used in image classification tasks, to evaluate the model’s classification with the following equations defined,


$$A\,c\,c\,u\,r\,a\,c\,y=\frac{T\,P+T\,N}{T\,P+F\,P+F\,N+T\,N}$$

$$P\,r\,e\,c\,i\,s\,i\,o\,n=\frac{T\,P}{T\,P+F\,P}$$

$$R\,e\,c\,a\,l\,l=\frac{T\,P}{T\,P+F\,N}$$

TP (true positive) indicates the positive samples predicted by the model as positive class, TN (true negative) indicates the negative samples predicted by the model as negative class, FP (false positive) indicates the negative samples predicted by the model as positive class, and FN (false negative) indicates the positive samples predicted by the model as negative class. Thus, the accuracy indicates the ratio of the number of samples correctly classified by the model to the total number of samples; the precision rate indicates the proportion of results predicted to be positive classes that are correct; and the recall rate indicates the proportion of samples that are actually positive classes that are correctly judged to be positive classes (Raschka, 2014).

## Procedures

### Alzheimer’s Disease Neuroimaging Initiative Data Acquisition

The Alzheimer’s Disease Neuroimaging Initiative (ADNI) was launched in 2003 and is led by Principal Investigator Michael W. Weiner, Ph.D., and its Web content is available at https://adni.loni.usc.edu/. The primary purpose of the ADNI is to investigate whether it is possible to combine a range of MRI, other biomarkers, and clinical and neuropsychological assessments to measure the progression of mild cognitive impairment and early AD. This project then opens up the data so that researchers worldwide can share data that can help explain the mechanisms of disease that occur in the preclinical and early stages of AD. Although there is currently no cure for AD, the ADNI database has dramatically facilitated the study of AD by researchers. In the ADNI database, data for each modality are classified into many categories, such as AD, MCI, and HC, according to their level of disease, and images of subjects with AD, MCI, and HC are classified using MRI data. However, the number of images in this study required a rather limited convolution neural network, and only a few slices of data are available for training and classification.

**FIGURE 7:**
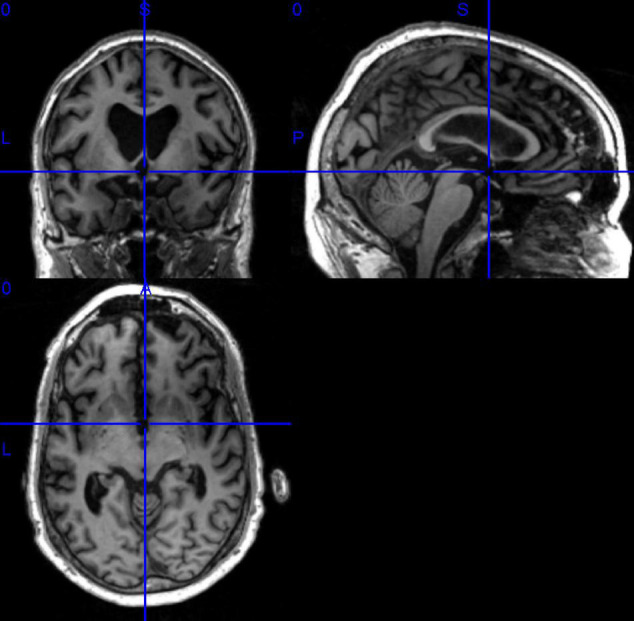
Example of 3D MRI image.

In this paper, we have selected data that are publicly available in the ADNI database. Before data download, ADNI will give a CSV table to record the subject’s number, gender, subject time, disease description, and other information for the data in this database Based on the table information, we filtered the MRI 3D image data with T1 weight from the ADNI1 period and got the data of 503 subjects, which contained 116 HC, 187 MCI, and 200 AD. It shows the statistical information of the final filtered data in Table 3. It showed the 3D MRI image of a randomly selected subject from the collated data in Figure 7.

**TABLE 3:** Detailed descriptive information of the filtered data.

Categories	Number of subjects	Age	Gender
AD	200	75 ± 7.9	118M/82F
MCI	187	77 ± 7.2	115M/72F
HC	116	77 ± 5.3	59M/57F

### Data Pre-processing

**FIGURE 8:**
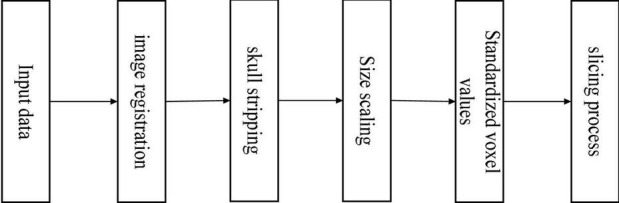
Flowchart of data pre-processing.

The data in the ADNI database may vary due to different sample acquisition conditions, which are mainly reflected in the equipment used to acquire the data which may be from other manufacturers, the operation of the medical personnel, the time of data acquisition, and so on. Various factors cause the differences between the data, so a series of pre-processing operations are required to achieve the requirements of feature extraction, feature selection, and image recognition and classification. It showed the steps of pre-processing the 3D MRI sample data in Figure 8.

We can divide the pre-processing of the data into the following five steps (Kun et al., 2020). (1) The downloaded data are in NIFTI format. It registered all MRI sample data with the MNI152 template using the tool FSL (Jenkinson et al., 2012) under Ubuntu, which is used to align the MRI data of all subjects to the same three-dimensional coordinate space to correct their spatial positions. (2) For a more accurate analysis of MRI sample data, it is necessary to use the skull culling method to remove voxel values from irrelevant background regions and keep only brain tissue in intracranial regions, reducing some irrelevant information and noise. Also, use the bet robust algorithm in FSL under Ubuntu for skull culling, set the fractional intensity threshold to 0.65, remove some unnecessary voxel values, and make image analysis more focused on image research. (3) Different MRI sample data have certain size differences. It is necessary to uniformly size the image after skull culling and scale it to 128 × 128 × 128. (4) Normalize the voxel value of the scaled MRI images, use the maximum and minimum normalization method, and then multiply it by the coefficient 255 to control the range of voxel value within 0–255. (5) With reference to other papers (Bae et al., 2020), the reason for selecting the 20 clearest coronal slices for each subject was to cover the entire hippocampus, as this region contains the most important information for the classification of AD. The processed data are sliced according to the coronal direction’s main view direction. We can regard the 3D MRI sample data as a stack of multiple continuous 2D images with a certain continuity. Therefore, 20 intervals with clear tissue structure in each sample are selected as the experimental data.

**FIGURE 9:**
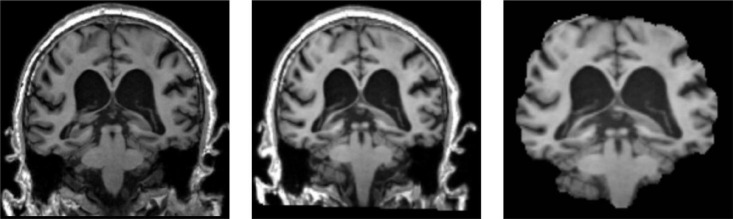
Sample slices of MRI images after registration and skull removal.

As shown in Figure 9, the 3D MRI sample data in the dataset were opened randomly using MRIcron software.^1^ The left picture is the original downloaded image from the database, the middle picture is the image after registration, and some pictures are the image after skull culling. When the score threshold parameter of skull culling is set to 0.65, more unnecessary parts are removed and only relatively clear parts of brain tissue are retained.

## Experimental Results

### The Analysis of Experimental Results

The experiments used 3D MRI data collated and filtered from the ADNI database, and the data were pre-processed and then sliced to get 10,060 MRI 2D slice data. The image data samples were then input into the proposed classification network to obtain the classification results for AD. It showed the results of the correct classification rate of MRI data in Table 4, and Acc stands for the classification accuracy, Pre stands for precision, Rec stands for recall. The bolded numbers in Tables 2–5 indicate the maximum values in their columns, and so on.

**TABLE 4:** Experimental results of AD:MCI classification on MRI slices (unit: %).

Classification task	Model	Accuracy	Precision	Recall
AD:MCI	VGG-16	91.84	91.62	91.62
	ResNet-18	93.66	94.44	86.29
	ResNet-50	95.21	92.46	93.40
	ResNet-50+Channel Shuffle	95.72	**95.74**	91.37
	CoT-ResNet-18 (ours)	95.72	93.43	93.91
	CCS-ResNet-50 (ours)	**96.23**	94.87	**93.91**

*Bolded numbers indicate the maximum value of the column.*

**TABLE 5:** Experimental results of AD:HC classification on MRI slices (unit: %).

Classification task	Model	Accuracy	Precision	Recall
AD:HC	VGG-16	93.29	92.65	85.52
	ResNet-18	94.01	92.09	93.20
	ResNet-50	94.98	93.28	94.40
	ResNet-50+Channel Shuffle	96.76	94.67	96.38
	CoT-ResNet-18 (ours)	94.34	92.49	93.60
	CCS-ResNet-50 (ours)	**97.90**	**96.47**	**98.40**

*Bolded numbers indicate the maximum value of the column.*

As shown in Table 4, compared with the traditional ResNet-18 model, the CoT-ResNet-18 model improves the accuracy of the AD:MCI classification task by 2.06%, indicating the effectiveness of the CoT module. On the basis of the ResNet-50 model, the Channel Shuffle mechanism is added, which has a slight improvement in performance. At the same time, the CoT model was added on the basis of the introduction of the Channel Shuffle mechanism and achieved a recognition accuracy rate of 96.23% in the classification task, with a precision rate of 94.87% and a recall rate of 93.91%.

As shown in Table 5, compared with the traditional ResNet-18 model, the CoT-ResNet-18 model improves the accuracy by 0.33% in the classification task of AD:HC. The CCS-ResNet-50 model achieved a recognition accuracy of 97.90% in this classification task, with a precision of 96.47% and a recall rate of 98.40%.

As shown in Table 6, compared with the traditional ResNet-18 model, the CoT-ResNet-18 model improves the accuracy of the MCI:HC classification task by 1.82%. The CCS-ResNet-50 model achieved a recognition accuracy of 91.75% in this classification task, with a precision rate of 92.98% and a recall rate of 94.40%.

**TABLE 6:** Experimental results of MCI:HC classification on MRI slices (unit: %).

Classification task	Model	Accuracy	Precision	Recall
MCI:HC	VGG-16	86.96	90.05	89.82
	ResNet-18	87.62	88.78	92.62
	ResNet-50	88.45	88.00	**95.17**
	ResNet-50+Channel Shuffle	90.76	**94.69**	90.84
	CoT-ResNet-18 (ours)	89.44	90.92	93.89
	CCS-ResNet-50 (ours)	**91.75**	92.98	94.40

*Bolded numbers indicate the maximum value of the column.*

As shown in Table 7, compared with the traditional ResNet-18 model, the CoT-ResNet-18 model improves the accuracy by 1.52% in the classification task of AD:MCI:HC. Compared with the traditional ResNet-50 model, the CCS-ResNet-50 model achieved a recognition accuracy of 88.61% in this classification task.

**TABLE 7:** Experimental results of AD:MCI:HC classification on MRI slices (unit: %).

Classification task	Model	Accuracy
AD:MCI:HC	VGG-16	84.38
	ResNet-18	86.79
	ResNet-50	87.30
	ResNet-50+Channel Shuffle	87.30
	CoT-ResNet-18 (ours)	88.31
	CCS-ResNet-50 (ours)	**88.61**

*Bolded numbers indicate the maximum value of the column.*

In summary, it showed the results to explain:

1. The effectiveness of CoT module. Compared with the traditional attention mechanism, the CoT module absorbs the contextual information among the nearest neighbors of the input information. Moreover, it combines the advantages of convolution operations to fuse the global and local information of the input features, which improves the expression ability of the output features and thus enhances the feature extraction ability of the residual blocks of ResNet.
2. The group convolution and Shuffle Channel mechanisms are introduced in the residual bottleneck block of ResNet-50, replacing two 1 × 1 convolution layers with group convolution and then enhancing the communication exchange of output features from different groups by random channel confusion. The experimental results show a slight reduction in the number of parameters of the model with guaranteed accuracy improvement.

### Comparison With Other Methods

Therefore, the comparison of the experimental results in this paper is consistent with previous studies that have used slices of MRI data from the ADNI to verify the performance of the algorithm.

As shown in Table 8, the two algorithms proposed in this paper have improved the classification results for different levels of Alzheimer’s disease compared with the methods proposed by previous researchers (Liu and Shen, 2014; Sarraf and Tofighi, 2016; De Luna and Marcia, 2021; Hasan et al., 2021; Xu, 2021), while this paper extends the classification between the two previously studied diseases to three categories and achieves better classification results. Among them, Sarraf et al. used the classical architecture LeNet-5 to classify functional MRI data of AD subjects with normal controls, and the accuracy of the tested data reached 96.85%, with the simple structure of LeNet-5 and limited ability to extract data. We further developed from the idea from Xu et al. whose approach was to use ResNet-50 as the backbone network and replace the 3 × 3 convolution with SKNet (Li et al., 2019), achieving a higher classification accuracy. Our idea is to replace the 3 × 3 convolution with a CoT module. The experiments showed that our experimental results achieved better classification results. Therefore, introducing the CoT module, group convolution, and Channel Shuffle mechanism into the residual block in ResNet is more reasonable and fully illustrates that the improved algorithm is competitive.

**TABLE 8:** Experimental results of classification of MRI data.

	AD:MCI			AD:HC			MCI:HC			AD:HC:MCI		
				
References	Acc	Pre	Rec	Acc	Pre	Rec	Acc	Pre	Rec	Acc	Pre	Rec
Ortiz et al.	84.00	–	79.12	90.09	–	86.12	83.14	–	67.26	–		
Luna et al.	–	–	–	–	–	–	78.90	79.39	78.49			
Liu et al.	86.30	–	84.55	93.08	–	92.67	87.24	–	85.55	–		
Sarraf et al.	–	–		96.85	–		–	–		–		
Xu et al.	95.30	–	94.50	97.18	–	94.92	89.53	–	88.67	–		
Hasan et al.	95.92	**96.00**	**96.00**	–	–	–	–	–	–	–	–	
CoT-ResNet-18 (ours)	95.72	93.43	93.91	94.34	92.49	93.60	89.44	90.92	93.89	88.31	–	–
CCS-ResNet-50 (ours)	**96.23**	94.87	93.91	**97.90**	**96.47**	**98.40**	**91.75**	**92.98**	**94.40**	**88.61**	–	–

*Bolded numbers indicate the maximum value of the column.*

## Discussion and Conclusion

### Discussion

The aim of this study is to investigate two novel models based on ResNet in order to classify AD, NC, and MCI, which can automatically extract features of MRI images for classification and can greatly simplify the pre-processing steps without complex manual processing operations. From the analysis of the experimental results in Table 4, the results showed that the application of the CoT module and the Channel Shuffle mechanism to ResNet can significantly improve the accuracy of the MRI image classification. We achieved satisfactory results in the image classification. In particular, in the classification task of AD:NC, a recognition accuracy of 97.50% was achieved, which was attributed to the more pronounced differences in the brain scans of structural MRI for HCs and AD patients, and the model was also able to accurately extract that feature and then use it for classification. From the comparison in Table 5, we also achieved good classification results in the classification tasks of AD:MCI, AD:HC, MCI:HC, and AD:NC:MCI, most of which were higher than the classification accuracies of the existing models. In summary, it is also shown that our proposed model has the following advantages: (1) The MRI images used do not require complex manual processing operations, such as segmentation of white matter and cerebrospinal fluid, and CoT-ResNet-18 and CCS-ResNet-50 models can automatically extract feature information from the more complex and subtle 2D images in the data; (2) the two models built using the ResNet-style architecture are reasonable for the analysis of medical images and they have a simple structure. It is noteworthy that both the CoT modules played a prominent role in enhancing the feature extraction of the residual blocks of ResNet, allowing the performance of the models to be further improved; and (3) we extended the traditional two-class classification of AD to three-class classification, and the results showed the excellent performance and robustness of the models. From the experimental results in Tables 4, 5, we can see that the two models used in this experiment have much room for improvement in improving the classification of the AD, MCI, and HC from each other. For future research, the focus can be on the optimization on the network structure, the change of convolution kernel, the step size of the convolution layer, and the update of the loss function. Of course, the above improvements to the models also apply to general image classification tasks, and perhaps, better results can be obtained.

### Conclusion

This paper designed two algorithms based on ResNet and showed their improved performance for AD image classification. First, the convolution layers of residual blocks are replaced with ResNet-18-based CoT modules, and the attention mechanism is introduced into the ResNet, where each residual block enhances the information extraction ability of the input data on top of the original one, thus improving the performance of the whole network. Second, based on the improvement in the ResNet-50 model, the 3 × 3 convolution layers in the ResNet-50 residual blocks are also replaced with CoT modules. The combination of the self-attention mechanism and convolution operations is used to extract the local and global information of the input information and fuse them to enhance the information extraction ability of the residual blocks. Then, two convolution layers are replaced by group convolution. We introduced the Channel Shuffle mechanism, which can randomly disrupt and reorganize the output feature maps after group convolution to enhance the information interaction between groups, reducing the number of model parameters without degrading the model performance. Finally, experiments are conducted on MRI slices ADNI data. Both algorithms designed in this paper can improve the classification accuracy compared with the existing conventional network model, showing the effectiveness of the ResNet-based attention module.

## Data Availability Statement

The original contributions presented in this study are included in the article/supplementary material, further inquiries can be directed to the corresponding authors.

## Author Contributions

QW: conceptualization, methodology, writing and editing, and financial and research support. CL: data processing, software, and writing. XL and BH: supervision, guidance, writing and editing, and financial and research support. All authors contributed to the article and approved the submitted version.

## Conflict of Interest

The authors declare that the research was conducted in the absence of any commercial or financial relationships that could be construed as a potential conflict of interest.

## Publisher’s Note

All claims expressed in this article are solely those of the authors and do not necessarily represent those of their affiliated organizations, or those of the publisher, the editors and the reviewers. Any product that may be evaluated in this article, or claim that may be made by its manufacturer, is not guaranteed or endorsed by the publisher.

## Footnotes

1
http://www.mricro.com/mricron
